# Notes and News

**Published:** 1900-11-03

**Authors:** 


### NOTES AND NEWS.

The new pavilion at the Royal Infirmary, Edinburgh, opened by the Princess Henry of Battenberg last week, is a Diamond Jubilee memorial. It is to be devoted to the treatment of women's diseases. The building is five storeys high. Three floors are devoted to the wards. The building contains besides a new lecture room, operating rooms, accommodation for nursing and domestic staff, and offices. Between the sanitary turrets at the end of each floor, and the wards, are open balconies. The basement is appropriated to medical baths for the use of the whole infirmary. The pavilion is connected with the main building by a covered corridor on the ground floor, and an open gallery on the first floor.

A new convalescent home for the County of Leicester has been added to the already existing Charnwood Forest Convalescent Home through the generosity of the Rev. W. H. Cooper, of Burleigh Hall, Loughborough. The home is to be called the Cooper Memorial Home, being erected in memory of the late Mrs. Cooper. It is placed upon an admirable site of two acres, part of which is natural woodland, part lawns and playgrounds. The building, which faces south, is to contain twenty-six children. Two large classrooms and four wards are provided for their use. One ward, with complete offices, is to serve as an isolation ward. The lavatories are shut off by cross ventilated passages, and cross ventilation is employed in the wards. The heating is by hot-water pipes and open fireplaces. The home has been presented completely furnished and equipped to the Committee of Management.

The Scotch methods of establishing charitable institutions are mostly more prudent than our own. It is not enough that the promoters should see their way clear to securing a site and a building, an endowment must be provided also. Two instances of this are now before us. The inhabitants of Perth have been offered a consumptive hospital more than a year ago by Sir Robert and Lady Pullar, but it appears unlikely it will be established just yet, as an endowment fund of £10,000 is regarded necessary, and a bazaar, as far ahead as September, 1901, is proposed, to augment the amount already collected, which equals less than half the sum required. Liverpool has just received the congratulation of Sir William Broadbent upon being the first city in the kingdom to erect a consumptive sanatorium, trusting to the importance of the work and its necessities to secure the necessary income for its maintenance.

Sir Christopher Nixon, M.D., has been elected President of the Royal College of Physicians of Ireland.

The *Kinfauns Castle* brought home the members of the South African Hospitals Commission on Saturday last.

Over 36,000 doses of diphtheria antitoxin have been issued from the laboratories of the Royal Colleges of Physicians and Surgeons during the past year.

The districts round Calcutta have been visited by an exceptionally heavy rainfall, and the general mortality has greatly risen. There has also been generally in India a marked increase of plague cases and a prevalence of cholera.

The "memorial" idea is carried rather far when it is proposed to raise a fund to endow a cottage hospital "In memory of the Passion Play of 1900" at Oberammergau. But no doubt there will be found plenty of people to join in the scheme. Some people will join anything.

Owing to the increase of the fee for the Diploma in Dental Surgery from 10 to 20 guineas, the income of the Royal College of Surgeons from this source has increased by £1,654. On the other hand, its income from fees for the conjoint examination has diminished to the extent of £1,094, as compared with the preceding year.

Three of the domestic staff of Charing Cross Hospital, Mary Ann Jones, Joseph Saunders, and Charles Taylor, have each received a certificate from the Royal Society for the Protection of Life from Fire, for their meritorious efforts to save the life of the girl who set fire to herself in August when lifting some beeswax and turpentine off the fire.

The Local Government Board have intimated that on the arrival of the steamship *Ben Lomond* in the Port of London from the Philippine Islands, on the 26th inst., a member of the crew was found to be recovering from suspected chronic plague. The malady has since been pronounced by Dr. Klein "to have been of the nature of plague." The man has been detained at the port hospital, and the vessel has been disinfected.

There is such a thing as "damning with faint praise," but in describing the consumption of liqueurs after dinner as a "physiologically correct proceeding" the *Lancet* has probably done more than this. It has deprived these beverages of their spice, so to speak. Many a young man takes a liqueur after meals, not because he likes it only, but because there is a certain up-to-date tone about the proceeding, which is destroyed by regarding it as a stomachic or corrective. It is reducing liqueurs to the level of peppermints.

The Harben Lectures of the Royal Institute of Public Health for 1900 will be given by Professor Calmette, M.D., Director of the Pasteur Institute at Lille, on Bubonic Plague, at the Royal College of Physicians on November 7th, 14th, and 21st, at 5 p.m. The Harveian Lectures will be given by Dr. Robert Maguire, M.D., on "The Prognosis and Treatment of Tuberculous Disease of the Lungs," at Stafford Rooms, Titchbourne Street, on November 1st, 8th and 15th. The Bradshaw Lecture will be given at the Royal College of Physicians by Dr. Archibald Garrod on November 6th, the subject being "The Urinary Pigments in their Pathological Aspects."

